# Towards the development of a screening tool to enhance the detection of elder abuse and neglect by emergency medical technicians (EMTs): a qualitative study

**DOI:** 10.1186/s12873-016-0084-3

**Published:** 2016-06-01

**Authors:** M. Brad Cannell, Katelyn K. Jetelina, Matt Zavadsky, Jennifer M. Reingle Gonzalez

**Affiliations:** Department of Biostatistics and Epidemiology, University of North Texas Health Science Center, Fort Worth, TX USA; University of Texas School of Public Health, Dallas Regional Campus, Dallas, TX USA; Department of Health Management and Policy, University of North Texas Health Science Center, Fort Worth, TX USA; MedStar Mobile Healthcare, Fort Worth, TX USA

**Keywords:** Elder abuse, Neglect, Older adult, Screening, Emergency medical services

## Abstract

**Background:**

To develop a screening tool to enhance elder abuse and neglect detection and reporting rates among emergency medical technicians (EMTs). Our primary aim was to identify the most salient indicators of elder abuse and neglect for potential inclusion on a screening tool. We also sought to identify practical elements of the tool that would optimize EMT uptake and use in the field, such as format, length and number of items, and types of response options available.

**Methods:**

Qualitative data were collected from 23 EMTs and Adult Protective Services (APS) caseworkers that participated in one of five semi-structured focus groups. Focus group data were iteratively coded by two coders using inductive thematic identification and data reduction. Findings were subject to interpretation by the research team.

**Results:**

EMTs and APS caseworks identified eight domains of items that might be included on a screening tool: (1) exterior home condition; (2) interior living conditions; (3) social support; (4) medical history; (5) caregiving quality; (6) physical condition of the older adult; (7) older adult’s behavior; and, (8) EMTs instincts. The screening tool should be based on observable cues in the physical or social environment, be very brief, easily integrated into electronic charting systems, and provide a decision rule for reporting guidance to optimize utility for EMTs in the field.

**Conclusions:**

We described characteristics of a screening tool for EMTs to enhance detection and reporting of elder abuse and neglect to APS. Future research should narrow identified items and evaluate how these domains positively predict confirmed cases of elder abuse and neglect.

**Electronic supplementary material:**

The online version of this article (doi:10.1186/s12873-016-0084-3) contains supplementary material, which is available to authorized users.

## Background

Older adults who experience abuse or neglect have 300 % greater risk of mortality and more chronic health problems, including depression or anxiety, chronic pain, high blood pressure and heart problems, than adults over 65 who are not abused or neglected [1–4]. These health conditions attributable to elder abuse and neglect increase healthcare expenditures by an estimated $5.3 billion annually [5].

The etiology of elder abuse and neglect is complex and appears to vary by abuse type [6–9]. For example, cohabitation and an established relationship with the perpetrator are risk factors for physical and emotional abuse [10, 11], but not financial exploitation [11]. In fact, victims of financial abuse tend to have known the perpetrator for only a short period of time and tend to live alone [6]. In many cases, the perpetrators of financial abuse tend to be the victim’s adult children [12]. Similarly, older adult victims of neglect tend to have cognitive impairments but not mental health conditions [6]. They also tend to have a greater burden of chronic disease and report greater levels of functional dependence upon others than those who do not experience neglect [6]. Victims of all forms of abuse (including physical, emotional, sexual abuse and neglect) have low social support with the exception of financial abuse [9]. Notably, physical and emotional abuse are most commonly reported to the police [9, 13]. Although a subset of older adults do experience multiple forms of victimization [7], these distinct etiologies make detection and intervention in cases of elder abuse, neglect and maltreatment difficult.

One of the primary challenges to fully understanding, and appropriately addressing elder abuse, neglect, and exploitation (EA) is under-detection and underreporting of its occurrence [9, 13–15]. For example, Acierno and colleagues found that roughly 11 % of older adults experience EA each year, but only 1 in 14 cases are reported to the authorities [9, 16, 17]. Similarly, results from one of the most comprehensive epidemiological studies on EA to date suggest that for every documented case of EA, 24 cases go unreported [13].

Because older adults are four-times more likely to use in-home emergency medical services than younger adults [18], EMTs are uniquely positioned to identify potential abusive or neglectful situations. EMTs can identify indicators of abuse or neglect (such as family interactions, home upkeep, medication availability, safety concerns and sanitation) not available to other emergency personnel, social workers or health care providers. In fact, during an investigation of the regional effects of EA underreporting, a representative from the region’s largest mobile healthcare (ambulance) service provider raised concerns over potential underreporting among area emergency medical technicians (EMTs). In 2013, this mobile healthcare provider responded to roughly 30,000 calls for older adults (aged 65+), but only reported 23 cases of EA to Adult Protective Services (APS) despite state-mandated reporting of suspected EA in Texas [19, 20]. Under-reporting is of particular concern because social isolation, dementia, and health and functional status are risk factors for elder abuse [21], and may hinder older adults’ ability to self-identify and self-report abuse or neglectful situations [22]. Alternatively, older adults with cognitive or functional limitations may fear retaliation by a family member or caregiver, and as a result, continue to live in abusive or neglectful situations. Concerns over this apparent under-reporting prompted the authors, who work closely with this mobile healthcare provider on a number of initiatives, to better understand the reasons for underreporting and opportunities to improve reporting rates by EMTs.

In a previous study authored by our research team, five general themes related to barriers to reporting EA among EMTs were identified. These themes included: (1) EMT apprehension towards violating older adults’ personal freedom to determine the conditions of their living environment; (2) EMT moral anxiety about the perceived negative consequences of an APS investigation on the older adult and/or their caregiver(s); (3) the time burden associated with reporting elder abuse or neglect to APS; (4) a perceived lack of case recall ability by EMTs, and, (5) low EMT confidence regarding ability to correctly identify potential elder abuse or neglect [23]. EMTs strongly believed that an appropriate EA screening tool would mitigate these barriers, and, as a result, increase the probability that they would report cases to APS [23]. In this study, we build on our previous investigation by identifying the most salient indicators of EA that such a screening tool should capture. Additionally, we will discuss the practical elements of the tool, such as format, length, and types of response options available, that could impact EMT uptake and use of the screening tool. In summary, our goal is to create a user-friendly screening tool with a small number of items that strongly predict confirmed cases of EA after investigation by APS.

## Methods

### Study design and setting

Specific details related to the study design and adherence to established qualitative research methods are provided in Table 1 [24]. A total of 23 EMTs and APS caseworkers were conveniently recruited to participate in a series of focus groups. EMTs (*n* = 11) were recruited from a large mobile healthcare provider in a major metropolitan area in North Texas. APS caseworkers (*n* = 12) were recruited from a regional APS office that serves the same metropolitan area. APS was included in this study because they are responsible for investigating any report of the abuse, neglect, or exploitation of an adult living with disability or an adult aged 65 or older, and if needed, provide services and take steps to prevent further harm. [25] Therefore, development of a screening tool to enhance reporting of abuse would be impossible without APS input.

**Table 1:** Consolidated criteria for reporting qualitative studies (COREQ) Checklist [24]

	Investigators (*n* = 2)	Graduate Research Assistants (GRAs; *n* = 4)
Domain 1: Research team and reflexivity		
* Personal characteristics*		
Interview/facilitator	At least one Investigator led each of the 5 focus groups	Three of the four project GRAs assisted in focus group administration as note-takers
Credentials	PhD	1) A doctoral candidate with a MPH degree
		2) A medical student
		3) M.S. student with experience in qualitative research
		4) Recent MPH graduate
Occupation	Assistant Professors at large research universities in the Dallas-Fort Worth area	All GRAs were current students during the time of the study
Gender	1 male; 1 female	1 male; 3 female
Experience and training	Both Investigators received a PhD in epidemiology from an accredited school of public health. One investigator has previously conducted and published qualitative research studies	All GRAs were required to read a training manual on qualitative research procedures. All GRAs had training in human subjects research
* Relationship with participants*		
Relationship established	No relationship with focus group participants before study commencement	
Participant knowledge of the interviewer	Participants had no knowledge of the researcher’s personal goals or reasons for doing the research before focus groups were conducted.	
Interviewer characteristics	Participants were informed that the Investigators were researchers from local universities. GRAs were introduced as research assistants. Participants were told that the focus groups were being conducted as part of a National Institute of Justice funded study to create a screening tool for EMTs that would attenuate barriers to reporting elder abuse and neglect.	
Domain 2: Study design		
* Theoretical framework*	EMTs	APS
Methodological orientation and theory	Grounded Theory	
* Participant selection*		
Sampling	Participants were sampled conveniently.	
Method of approach	All EMTs employed by the mobile healthcare provider and APS caseworkers were e-mailed by executive staff members at each agency (not the research team).	
Sample size	11	12
Non-participation	Executive staff members at the mobile healthcare provider and APS were responsible for recruiting participants. Given the sensitivity of this topic, the research team was not provided identifiable information about the participants (or potential participants) and information about non-participation could not be assessed.	
* Setting*		
Setting of data collection	Mobile healthcare provider office	Local APS branch office
Presence of non-participants	No persons other than the researchers and the participants were present during data collection	
Description of sample	Gender: 7 were men and 4 were women.	Gender: 11 were women, 1 man
	Race/Ethnicity: All were White, and one also identified as Hispanic.	Race/Ethnicity: One participant was White and the remainder were Black.
	Age: Mean was 40 years old (range 20–67)	Age: Mean of 39 years (range 23–63)
	Experience: Mean paramedic-level EMT for 7 years (range 2–22 years)	Experience: APS employee for 10 years (range <1–35)
* Data collection*		
Interview guide	The authors provided questions and prompts. However, the focus groups were semi-structured in nature and the conversation commonly deviated from the script.	
Repeat interviews	No repeat interviews were carried out.	
Audio/visual recording	Audio, but not visual, recording was used to collect data. After recording were transcribed by a GRA and verified by an Investigator, recordings were destroyed.	
Field notes	The secondary interviewer took field notes during each focus group.	
Duration	1–1.5 h	
Data saturation	The research team discussed data saturation after the first 3 focus groups and again after 2 additional focus groups. Data collection continued after the first 3 focus groups because the transcripts did not reflect saturation (new themes were being identified in focus group 3). After 5 focus groups, data collection was deemed complete, as no new themes were identified after transcript examination.	
Transcripts returned	Transcripts were not returned to participants for comments or corrections, as no identifiable information about participants was collected.	
Domain 3: Analysis and findings		
* Data analysis*		
Number of coders	Two coders coded data (one Investigator and one trained GRA)	
Description of the coding tree	There was no a priori coding tree created due to the limited theoretical knowledge base in this area. The two coders used a ‘two rivers’ approach to coding and identifying themes [43]	
Derivation of themes	Themes were derived from the data and not identified in advance	
Software	Dedoose 2.0 was used for data management	
Participant checking	Participants did not provide feedback on the findings. However, executive staff members at the mobile healthcare provider organization were provided a list of major themes.	
* Reporting*		
Quotations presented	Participant quotations are presented to illustrate themes.	
Data and findings consistent	There was consistency between the data presented and study findings.	
Clarity of major themes	All major themes relevant to the research question are discussed.	
Clarity of minor themes	Minor themes/diverse cases are discussed where relevant in the text.	

At each site, administrators (study partners) e-mailed all employees (APS caseworkers and EMTs) with an invitation to participate in this study. The invitation made clear that participation was voluntary, and that choosing not to participate would not impact their employment in any way. Study partners scheduled focus groups, and participants were paid their usual wage by their agency for the duration of the focus group. Study partners were not involved in data collection, and only research personnel not affiliated with either agency hosted, transcribed, coded and analyzed focus group data.

### Methods and measurements

Focus groups were conducted on-site at each agency’s location between May and June of 2015. When participants arrived, informed consent was obtained and participants completed a brief demographic questionnaire. Participants were informed that the purpose of these focus groups is to understand EMT experiences regarding EA, barriers that might exist to reporting elder abuse, and identification of environmental (social and physical) cues indicative of EA that can be used in the development of a screening tool for use by EMTs. To minimize social desirability biases, participants were instructed that there were no right or wrong answers, and that participants’ identifiable information will not be linked with their responses in any way. Sessions began with a general discussion about participant experiences with reporting EA (for EMTs) or working with EMTs who previously reported EA (for APS) [26].

Next, participants were given a list of EA screening items from existing screening tools that were developed for other groups (e.g., researchers or physicians; see Additional file 1) [27–31]. Participants were asked to discuss their general feelings about this list of screening items and to provide specific feedback regarding the potential utility of each item in helping EMTs identify and report potential cases of EA. Moderators used probing questions to facilitate rich discussion about how these items might be used, adapted or modified for EMTs. To prevent loss of data, notes were taken during the focus group sessions by a member of the research team. To protect anonymity, participants were assigned a number to use instead of their name during the focus group sessions. The Institutional Review Board at the University of North Texas Health Science Center approved the data collection protocol for these focus groups.

### Analysis

Systematic procedures of qualitative data analysis included: intensive reading of the text and group discussion of the transcripts by all members of the research team, coding by two investigators, inductive thematic identification, data reduction, and interpretation. These processes were iterative and coding occurred during the same time period for both coders (May-July, 2015). Coding for each focus group was initiated immediately after transcription and **i**nconsistencies in the coding process and findings were resolved by the research team. During this iterative coding and recoding process, the research team concluded that saturation was achieved when all themes identified in the fifth focus group were redundant with themes identified during the previous four focus groups [32]. Dedoose was used for all coding, organization and thematic analysis [33].

## Results

### Characteristics of study subjects

Characteristics of the focus group participants are provided in Table 2. Ninety-one percent of EMTs made at least one report of suspected EA to APS during their tenure, and all EMTs expressed a desire to work more closely with APS and believed that they have a “*a vicarious responsibility [to report suspicions of EA]”*

**Table 2:** Selected characteristics of EMTs and APS caseworkers who participated in focus groups about elder abuse and neglect, April – May 2015

Characteristic	Total (*n* = 23)	EMT (*n* = 11)	APS (*n* = 12)
	n (percent)		
Sex			
Male	8 (35 %)	7 (64 %)	1 (8 %)
Female	15 (65 %)	4 (36 %)	11 (92 %)
Age			
20–29	5 (22 %)	4 (36 %)	1 (8 %)
30–39	8 (35 %)	1 (9 %)	7 (58 %)
40–49	6 (26 %)	4 (36 %)	2 (17 %)
50 and older	4 (17 %)	2 (18 %)	2 (17 %)
Race			
White, non-Hispanic	11 (48 %)	10 (91 %)	1 (8 %)
Black, non-Hispanic	11 (48 %)	0 (0 %)	11 (92 %)
Hispanic, any-race	1 (4 %)	1 (9 %)	0 (0 %)
Married	13 (57 %)	6 (55 %)	7 (58 %)
College Graduate	15 (65 %)	3 (27 %)	12 (100 %)
Length of employment			
Less than 5 years	7 (30 %)	3 (27 %)	4 (33 %)
5 to less than 10 years	8 (35 %)	3 (27 %)	5 (42 %)
10 years or more	4 (17 %)	1 (9 %)	3 (25 %)
Ever provided care or assistance to a friend or family member	11 (48 %)	4 (36 %)	7 (58 %)

### Main results

#### EMT and APS-identified indicators of EA

The primary aim of this study was to identify the most salient indicators of EA that might be included on a screening tool. Identified indicators were highly consistent in all five focus groups. Eight domains of EA indicators were identified: 1) condition of the home exterior, 2) condition of the inside home environment, 3) social support, 4) medical history and medications, 5) caregiving indicators, 6) physical condition of the older adult, 7) behavior of the older adult, and 8) the EMT’s own “gut” instincts.

1. *Condition of the Outside Home Environment*

Access to the home and to the older adult within the home was identified as a potential indicator of EA. Specifically, EMTs and APS caseworkers have suspected abuse or neglect when older adults are restricted to a single living space within the dwelling, most commonly in the back of the home. In the most serious circumstances, “*[the patient was staying in a] shed in the backyard with a bed in it,” “[the patient is] situated [in the backyard] … you wouldn’t even put your dog back there.” or, “[the caregiver] chained their grandmother in a room in the back [of the house].”* Further, EMTs and APS caseworkers noted that they tend to associate an “unkept” living environment with abuse and neglect. For instance, participants stated that they might suspect abuse or neglect in a home where “*everything is overgrown, weeds are tall, shrub cover the entrances [to the home], and… mail piling up,” “trees and lots of branches [are] everywhere,”* and the *“lawn is not mowed.”*

2) *Condition of the Inside Home Environment*

The most consistently identified correlates of EA were reflective of the inside home environment. EMTs and APS caseworkers reiterated that the presence of clutter raised their suspicions of abuse or neglect:

“*… you see the dirty dishes, the place is just in disarray; what I mean about that is that there is just a lot of clutter… their insulation is … stacked [next to] the walls.”*

In some cases, emergency personnel could “*barely get inside the door,”* and when they gained access, trash or household possessions were “*scattered all over the place”* to the point where clutter posed a fall risk for the older adult. The words “hoarding” and “hoarder” were commonly used in every focus group, even though the semi-structured questionnaire did not include a reference to this term.

Participants associated cases of EA with general (and in many cases, severe) household disrepair and neglect. EMTs identified disrepair as, “*living rooms with two inches of standing water,*” or *“[the older adult] does not have a heater in the house…[because] they are trying to save money.”* In addition, both APS caseworkers and EMTs noted that bugs and cockroaches are commonly present, and they might notice, “*roach poop… piled up in the corner,”* or “*roaches crawling all over the walls.”* Large rodents and bed bugs may also be present and/or visible in the home.

3) *Social Support*

Participants noted that the absence of social support might reflect abuse. This social support may be in the form of familial support, peer support, or participation in social functions (“*Find out… if they have friends, if they participate in any groups, those types of things. They kind of let you know if someone is checking in on them, or helping them in any way to help us better determine if they are indeed safe at home, if there is anyone looking in on them”*). Lack of any inter-personal network or participation in social gatherings was identified as indicator of abuse or neglect, particularly when the older adult’s family was disengaged (“*kids not coming to see them for years”*).

Additionally, participants identified third-party referrals or calls to EMS as an indication that further investigation into a situation is warranted. EMTs reported that friends of the older adult and neighbors commonly call EMS because they are concerned about the older adult’s condition. Although these peers do not live in the home, they may call repeatedly in an attempt to get help from EMS even when no immediate medical concern is present. In other words, friends and neighbors perceive that EMTs will view the situation as problematic and notify the appropriate department (e.g., APS) to help.

4) *Medical History and Medications*

Inadequate care for medical conditions directly reflects quality of care in the home and in many cases, could constitute medical neglect. EMTs might notice that an older adult’s medications are not being filled in a timely manner, that multiple physicians prescribe the same medications, that a patient is taking expired medication, or that “*multiple medications are mixed in the same bottle… two or three different colored pills in the same bottle.*” In other cases, an older adult with diabetes may report not having transportation to a dialysis clinic, or may be unable to locate their glucometer or insulin to manage their condition. Although these examples may constitute medical neglect, they may also represent poor quality caregiving in the home.

5) *Caregiving Indicators*

EMTs reported that many older adults have a caregiver or family member charged with managing the older adult’s needs. Although a caregiver may be present, the level of care provided varies. This discordance in the quality and frequency of caregiving might be detected by EMTs. One APS caseworker provided the following example of how caregiver reports do not support the findings from their investigation or the older adult’s report, thus leading them to suspect abuse or neglect:

“*[the older adult] hasn’t eaten in 3 days and the caregiver responds, ‘But I cook for her all the time ma’am, like I make her these big meals and she usually loves to eat but she’s just not eating now,’ and man as soon as y’all get her up, I mean she was on the floor on a cushion that was saturated in urine, like it was starting to disintegrate, that’s how bad the cushion was.”*

In other cases, caregivers are simply absent, or the older adult lives with family members or other caregivers who are providing inadequate care. For example, a caregiver might not be providing for an older adult’s basic needs, such as changing diapers, providing clothing and bed sheets, providing food and water, helping the older adult use the bathroom or bathing, turning bed-bound adults, or adhering to a medication regimen. The absence of family and caregivers was consistently noted as a strong indicator of abuse or neglect.

EMTs also noted that caregiver drug use, drug dealing, or apparent alcohol problems, identified by alcohol bottles or drug paraphernalia in the home, visible “track marks” or EMT-perceived caregiver impairment, may indicate neglect or at the very least, absence of quality care provision. Although evidence of alcohol or drug use alone may not qualify as abuse or neglect, EMTs view these situations as red flags that, in conjunction with other factors, might justify a report to APS.

Finally, a common theme related to EA identified by both APS caseworkers and EMTs was caregiver presence and demeanor. Two extremes were identified as potentially problematic: 1) family or caregiver hovering around APS or emergency personnel, failing to allow an older adult/patient to answer questions on their own (“*hovering and being overprotective and answering questions for [the patient],” or “the relatives were overly helpful, almost like [they were] trying to mask something”)*; and 2) the family member/caregiver may have no idea what is going on, be unaware of the older adult’s medical needs, and generally disengaged from the situation.

6) *Physical Condition of the Older Adult*

EMTs and APS caseworkers consistently associated abuse and neglect with a bad odor (“body odor,” ammonia/urine, and other smells) and poor hygiene, inappropriate clothing, and patient mental health (particularly, dementia). Further, all participants perceived older adults who are unable to care for themselves, or are bed bound, as particularly vulnerable. Many injuries, including layered bruises, black eyes, and untreated broken bones, indicate abuse and neglect to EMTs. One EMT noted,

*“[abuse might be indicated when a patient] has varying levels of bruising, or you see many old breaks and they were a housewife their entire career, and their hands and forearms look like breaks that didn’t mend, you’re not really in the middle of the country where they might not let them reset. But just walking into the house, something [is] just not right…”*

Some injuries or medical problems, such as chronic sepsis, ulcers, rashes, urine burns, and dehydration, are manifestations of abusive or neglectful situations.

7) *Behavior of the Older Adult*

EMTs noted that they suspect an abusive situation when patients “*hesitate to answer [their questions],*” avoid eye contact, and most importantly, when the patient’s behavior and demeanor changes in the presence of the caregiver. In these cases, patients appear atypically nervous or fearful to the EMT, refuse to answer EMT questions in front of the caregiver, or suddenly become guarded in front of the caregiver when they were previously very open with the EMT. Some EMTs acknowledged that these indicators may be linked with cultural norms (specifically, failure to make eye contact), fear attributable to their illness or an injury, or fear of being placed in a nursing home, rather than abuse.

8) *EMT “Gut” Instincts*

Finally, some of the most salient findings suggested that EMT “gut” instincts may lead them to suspect abuse or neglect. EMTs mentioned,

“*[You get this] gut feeling, it’s like something is not right in this house, something not right I can’t put my thumb on it, but something bad is happening here.”*

*“Take in the big picture, if there is that feeling something’s wrong, then usually there is something wrong.”*

*“If it looks bad, it probably is bad,”* and one EMT noted, *“you smell a skunk when you step on it.”* Overall, these passages suggest that experienced EMTs develop a sense that leads them to suspect an abusive or neglectful situation, even if they are not able to articulate or rationalize their suspicion.

#### Characteristics of an optimally designed screening tool

If a screening tool was available for use by EMTs, the tool must be incorporated into their electronic medical record system, and should not be paper-based to maximize use and accessibility. In light of time restrictions, EMTs suggested that the tool be very brief and include 5 to 10 (yes/no) items, and take less than 10 min to complete. These items should not require them to question older adults or caregivers. When EMTs respond to a call, their first priority is the medical emergency that prompted the call; therefore, EMTs should be able to answer each question in the screening tool based upon observable characteristics of the environment, patient, caregiver, and context. Furthermore, a well-designed tool should provide an objective indication of risk. In Texas and other states with mandatory reporting laws, it would be inappropriate for the tool to direct an EMT *not* to report EA; however, the tool could assign risk categories to the situation (e.g., low risk/high risk) based upon the number and combination of indicators present. EMTs believed that an objective assessment provided by the screening tool would alleviate the moral anxiety currently faced when they make a “judgment call” to report (or not report) a potential case to APS. EMTs agreed that they would use a tool that met these criteria if it was available. APS caseworkers also strongly supported the development of this tool with the perception that it would enhance the quality of data received from EMTs.

## Discussion

This study identified eight domains of indicators that may be observed by EMTs during calls for service to begin to address the problem of EA under-reporting. The domains that should be considered for inclusion on such a tool include: (1) the condition of the outside areas around the home; (2) conditions inside the home; (3) the presence/adequacy of social support; (4) medical history and medication use/misuse; (5) caregiving indicators; (6) the physical condition of the older adult; (7) the older adult’s behavior; and, (8) EMTs’ instincts.

These domains are generally consistent with the literature on risk factors for EA [11, 16, 34, 35], with the exception of EMT instincts. A study of elder maltreatment in New York identified poverty and caregiver disengagement as the only two indicators of abuse or neglect that could be identified by third parties, such as EMTs [13]. Lachs et al. identified other domains of abuse noted in this study, including functional and cognitive impairment, low overall social support and fewer social ties [16]. Lachs also found that older adults who live alone are less likely to be abused or neglected than those who live with family or a caregiver [16]. There is disagreement in the literature on this topic, as data from the National Crime Victimization Survey suggest that those who live in a single person household were up to five times more likely to be victimized than those who live with a spouse or a spouse and children [36].

Our second aim was to identify the characteristics of an EA screening tool that were likely to affect its utility in the field. Our results suggest that the screening tool should: (1) be easily integrated into their current workflow—including their electronic charting systems; (2) be very brief (5–10 items); (3) be based on observable cues in the physical / social environment; (4) not necessitate directly questioning the older adult or caregiver; and, (5) provide a risk score or reporting guidance to alleviate moral anxiety associated with making decisions to report a suspected case to APS [23].

Several screening tools for EA currently exist, but none to our knowledge are appropriate for use by EMTs in their current form [27–29, 37–40]. For example, some existing tools require that questions be asked of the caregiver and/or the older adult [31]. If a caregiver is not present when EMTs enter a residence, these tools that require a caregiver response could not be completed. Other tools were designed for physicians and are simply not practical for prehospital care, field-based settings [27, 29]. In the field, EMT’s goal is to provide medical care; detection of elder abuse is ancillary. However, it is important to note that existing screening tools, such as the Elder Abuse Suspicion Index (EASI) [29], incorporate clinical judgement into the final assessment (equivalent to our ‘gut instinct’ item). Finally, other tools are very lengthy and cannot reasonably be completed in a field setting [41]. The limitations of previous developed screening tools highlight the need for a validated tool that relies upon EMT’s contextual observation rather than questionnaires.

Importantly, Lachs and Pillemer suggest that screening tools designed to detect elder abuse are limited in that older adults and their caregivers may actively attempt to hide symptoms of elder abuse from the person administering the screening tool [35]. In their review, Lachs and Pillemer suggest that older adults may wish to hide symptoms of victimization from a physician when visiting a doctor’s office [35]. This highlights the utility of in-home observation by EMTs, as medical emergency calls for service provide little time for caregivers or older adults seeking to hide visible symptoms of elder abuse in their home.

It is also important to note that any tool designed to detect elder abuse or guide EMT’s reporting practices is *not* diagnostic in nature. EMTs should always be trained to use their own judgement and report elder abuse when suspected, regardless of any risk score. However, given the very low rate by which elder abuse is reported, we expect that the risk score or reporting guidance provided by this tool will increase the frequency by which cases of elder abuse are detected and referred to APS for investigation.

### Limitations

These results should be considered in light of several imitations. First, data were collected from a small number of EMTs and APS caseworkers who were geographically limited to one region in North Texas. Therefore, our findings may not generalize to EMTs in other regions or to other professionals who may also see a need for a similar tool (e.g., police, firefighters, other social service providers). Additionally, researchers who conducted the focus groups may have introduced information bias through the probing process, although probing is common practice in qualitative research [42]. Finally, while EMTs and APS caseworkers agreed on the eight general domains that indicate EA, this study did not capture information about the specific screening items that best measure these domains.

## Conclusions

Despite these limitations, this is the first study to provide formative data about the need for, and design of, a screening tool specifically for EMTs. The successful development and deployment of an EA screening tool has the potential for broad public health impact. Because the practice of under-reporting is likely not limited to one city in Texas, there may be millions of similarly missed opportunities to detect EA nation-wide each year. Additionally, the support and access we received from leadership at the mobile healthcare provider and APS allowed for an uncensored discussion of a sensitive subject. The results of the current study provide evidence that EMTs do, in fact, regularly interact with older adults who are living with risk factors for abuse, and that they desire a screening tool to help them overcome current barriers to reporting. This information enabled us to conduct a unique study that moves the field towards one potential solution that could help address the underreporting of EA. Future research should seek to develop and validate a tool to enhance detection and reduce the underreporting of EA. This will not only benefit EMT and APS investigations, but ultimately improve the lives of older adults.

## Abbreviations

APS, Adult Protective Services; EA, elder abuse, neglect, and exploitation, EMTs, emergency medical technicians

## Additional file

Additional file 1: Example Elder Abuse Screening Instrument Items. (DOCX 27 kb)
